# Lung Ultrasound Efficacy in Monitoring Post-SARS-CoV-2 Pneumonia and Inflammatory Biomarkers in Pediatric Patients

**DOI:** 10.3390/medicina60081296

**Published:** 2024-08-11

**Authors:** Ramona Chelcea, Mihaela Dediu, Diana Dabica, Sorina Maria Denisa Laitin, Ioana Mihaiela Ciuca

**Affiliations:** 1 Doctoral School, “Victor Babes” University of Medicine and Pharmacy, Eftimie Murgu Square 2, 300041 Timisoara, Romania; ramona.chelcea@umft.ro (R.C.); diana.popin@umft.ro (D.D.); 2 Department of Pediatrics, “Victor Babes” University of Medicine and Pharmacy, Eftimie Murgu Square 2, 300041 Timisoara, Romania; dediu.mihaela@umft.ro (M.D.); ciuca.ioana@umft.ro (I.M.C.); 3 Discipline of Epidemiology, Victor Babes University of Medicine and Pharmacy, 300041 Timisoara, Romania

**Keywords:** pediatrics, pediatric disease, SARS-CoV-2

## Abstract

*Background and Objectives*: Recognizing the crucial gaps in our understanding of pediatric pneumonia post-SARS-CoV-2 infection, this study aimed to assess the relationship between Pediatric Pneumonia Ultrasound Scores (PedPne) and inflammatory biomarkers. The primary objective of this study is to evaluate the predictive value of PedPne in comparison with inflammatory biomarkers (IL-6 and dNLR) for the development of pneumonia in pediatric patients following SARS-CoV-2 infection. *Materials and Methods*: This longitudinal observational study collected data from pediatric patients diagnosed with pneumonia after an acute SARS-CoV2 infection. The study focused on analyzing changes in PedPne scores and inflammatory markers such as IL-6 and dNLR from initial admission to follow-up at 7 days. Statistical analysis involved calculating the sensitivity, specificity, and Area Under the Curve (AUC) for each biomarker, alongside regression analysis to determine their hazard ratios for predicting pneumonia development. *Results*: The analysis identified significant cutoff values for dNLR at 1.88 (sensitivity 77.0%, specificity 85.7%, AUC 0.802, *p* < 0.001), IL-6 at 6.1 pg/mL (sensitivity 70.3%, specificity 92.9%, AUC 0.869, *p* < 0.001), and PedPne score at 3.3 (sensitivity 75.7%, specificity 78.6%, AUC 0.794, *p* < 0.001). Conversely, NLR showed lower diagnostic performance (AUC 0.485, *p* = 0.327). Regression analysis further highlighted the strong predictive power of these markers, with IL-6 showing a fourfold increase in pneumonia risk (HR = 4.25, CI: 2.07–9.53, *p* < 0.001), dNLR indicating more than a twofold increase (HR = 2.53, CI: 1.19–6.97, *p* = 0.006), and PedPne score associated with more than a doubling of the risk (HR = 2.60, CI: 1.33–5.18, *p* < 0.001). *Conclusions*: The study conclusively demonstrated that both PedPne ultrasound scores and specific inflammatory biomarkers such as dNLR and IL-6 are significant predictors of pneumonia development in pediatric patients post-COVID-19 infection. These findings advocate for the integration of these biomarkers in routine clinical assessments to enhance the diagnostic accuracy and management of pneumonia in children following SARS-CoV-2 infection.

## 1. Introduction

Recent studies have underscored the heightened risk of developing secondary bacterial pneumonia after a primary viral infection such as SARS-CoV-2 [1,2]. This type of pneumonia, often severe, necessitates a better understanding of its pathogenesis and diagnostic markers. Reports indicate that approximately 15% of hospitalized COVID-19 patients develop secondary pneumonia, characterized by persistent fever, worsening respiratory symptoms, and new infiltrates on chest imaging, which complicates the clinical course and can significantly extend hospital stay and increase mortality [3,4,5].

Chest ultrasound has emerged as a vital tool in pediatric pneumonia, offering a radiation-free alternative to traditional imaging techniques like chest X-rays and CT scans [6,7]. Its application has proven particularly valuable in assessing lung involvement and guiding treatment decisions in real time. The Pediatric Pneumonia Ultrasound Score (PedPne) is a quantifiable measure that has been correlated with clinical outcomes, allowing clinicians to monitor disease progression and response to treatment with greater precision [8].

Inflammatory biomarkers also play a crucial role in the diagnosis and management of infectious diseases, including pneumonia. Markers such as C-reactive protein (CRP), procalcitonin, and interleukin-6 (IL-6) have been widely studied for their ability to indicate the presence and severity of infection, where elevated levels have been associated with worse outcomes in respiratory infections, reflecting the intensity of the inflammatory response triggered by pathogens [9,10,11].

Despite the established roles of ultrasound scoring and inflammatory markers, there is a notable gap in longitudinal studies that integrate these parameters in the context of post-SARS-CoV-2 pneumonia [12,13]. Furthermore, there is an important focus in examining the correlation between ultrasound findings and inflammatory markers over the course of the illness and during recovery [14,15]. As such, an integrated approach utilizing both PedPne ultrasound scores and inflammatory biomarkers could provide a comprehensive assessment of pneumonia’s impact and progression, potentially leading to more tailored and effective treatment strategies.

Given the critical gaps in our understanding and the potential of ultrasound and biomarker integration, this study hypothesizes that a significant correlation exists between PedPne ultrasound scores and inflammatory biomarkers in pediatric patients developing pneumonia post-SARS-CoV-2 infection. The objectives of this study are to analyze the changes in PedPne scores and inflammatory markers from the initial presentation to the subsequent follow-up at 7 days of treatment, and to evaluate how these measurements correlate with pneumonia development. Pediatric pneumonia post-SARS-CoV-2 infection poses unique diagnostic challenges due to its variable symptoms and overlap with other respiratory infections, often leading to delayed treatment [16,17,18]. This study addresses these challenges by assessing the utility of PedPne and inflammatory biomarkers, aiming to enhance early diagnosis and management. Integrating these tools into clinical practice can improve diagnostic accuracy, enable timely interventions, and ultimately reduce morbidity and healthcare burdens associated with pediatric respiratory infections.

## 2. Materials and Methods

### 2.1. Study Design and Ethics

This longitudinal observational study evaluates the correlation between PedPne and inflammatory biomarkers in pediatric patients who developed pneumonia following SARS-CoV-2 infection. The study cohort comprises patients evaluated in outpatient settings at Pediatric Pulmonology Ward of the Pediatric Clinic, Clinical County Hospital Timisoara, between September 2022 and April 2024, presenting pneumonia signs, who had previously been diagnosed with SARS-CoV2 infection. The research protocol involved collecting data at initial presentation for hospital admission and during follow-up visits at 7–10 days of treatment. Data collection was carried out through both electronic health records and direct clinical assessments.

This study has received approval from the institutional Ethic Committee (number 461/18.04.2024), of Clinical County Hospital Timisoara adhering to the ethical standards set by the Declaration of Helsinki and the EU Good Clinical Practice Directive (2005/28/EC). All patient information was anonymized prior to analysis to ensure confidentiality. Additionally, written informed consent was obtained from the legal guardians of all participants, confirming compliance with ethical requirements.

### 2.2. Inclusion and Exclusion Criteria

Inclusion criteria for the study required participants to have a confirmed diagnosis of SARS-CoV-2 infection, established through RT-PCR testing, and subsequent development of pneumonia, identified through clinical and ultrasound findings. All participants were pediatric patients admitted to the outpatient or inpatient services of the pediatric clinic during the specified study period. Eligibility also depended on the availability of comprehensive medical records, including detailed ultrasound evaluations, inflammatory biomarker levels (e.g., CRP, procalcitonin), and clinical outcomes.

Exclusion criteria included patients who did not develop pneumonia following SARS-CoV-2 infection, those who had pre-existing lung diseases that could confound the ultrasound and biomarker results, and patients whose legal guardians did not provide written consent for participation in the study. Additionally, patients with incomplete data sets, such as missing follow-up ultrasound scores or biomarker results, were excluded to maintain the integrity of the research findings.

### 2.3. Data Collection and Variables

For our study, we systematically collected and analyzed variables from pediatric patients hospitalized with COVID-19, focusing on differences between those with and without pneumonia. The primary demographic variable was age, categorized into three groups: 1–6 years, 7–12 years, and 13–18 years. Additionally, body mass index (BMI) and vitamin D levels were measured to explore their potential influence on the disease severity. We included clinical and laboratory data, assessing initial and subsequent changes at 7 days post-hospitalization. Key inflammatory markers were evaluated, including C-reactive protein (CRP), procalcitonin, interleukin-6 (IL-6), white blood cell (WBC) count, percentages of neutrophils, and erythrocyte sedimentation rate (ESR). Ferritin levels and derived neutrophil-to-lymphocyte ratio (dNLR) were also calculated to provide insights into the systemic inflammatory response. Additionally, previously data on SARS-CoV2-specific immunoglobulins M and G were collected from patients’ data. General immunoglobulin levels (IgG, IgM, IgA, IgE) were measured in patients with pneumonia. Pediatric pneumonia severity was quantitatively assessed using the Pediatric Pneumonia Ultrasound Score (PedPne US) at first evaluation and after 7 to 10 days to monitor progression or resolution of pneumonia.

In the study protocol, lung ultrasound was performed at admission by a pediatric pulmonologist proficient in LUS techniques, using a Venue Go™ by GE HealthCare (Chicago, Illinois, USA) ultrasound system with both linear (7–12 MHz) and convex (3.5–5 MHz) probes. The ultrasound examination involved scanning through longitudinal and transversal sections across various anatomical lines and included a transabdominal approach for deeper insights. The lung was sectioned into six areas for detailed analysis, and specific ultrasound artifacts like the presence of consolidation and pleural effusion were quantified. The diagnostic criteria included hypoechoic images erasing A-lines, dynamic bronchograms, and B-line patterns with specified points assigned based on the severity and nature of the findings in the PedPne score.

The PedPne score, a systematic tool for assessing pediatric pneumonia severity using lung ultrasound, is quantified based on specific ultrasound findings reflecting lung pathology [19]. The score incorporates several diagnostic features: perilesional B-lines (more than three B-lines per intercostal space score 1 point and coalescent B-lines score 2 points), varying sizes of lung consolidations (non-translobar consolidations smaller than 2 cm score 3 points, larger than 2 cm score 4 points, and translobar consolidations score 5 points), and pleural effusions (simple pleural effusion scores 6 points, complicated nonseptated pleural effusion scores 7 points, complex septate pleural effusion scores 8 points, and homogenous hyperechoic effusion or empyema scores 9 points). Each of these elements is assessed during a detailed lung ultrasound, where each hemithorax was divided into six areas, and each lung feature was scored according to its severity and diagnostic impact on pediatric pneumonia.

### 2.4. Statistics

Data management and analysis were carried out using the statistical software R version 4.0.3. A sample size of minimum 62 patients was calculated as being sufficient for the current study. Continuous variables were presented as means ± standard deviation (SD), while categorical variables were summarized using frequencies and percentages. To compare the means of continuous data across groups, the study employed Student’s *t*-test, whereas categorical data comparisons were made using the Chi-square test. For the key laboratory markers under study, the optimal cutoff values were determined using receiver operating characteristic (ROC) curves, from which the sensitivity, specificity, and Area Under the Curve (AUC) were calculated to evaluate their predictive power. Additionally, regression analysis was conducted to ascertain the hazard ratios for pneumonia development based on the biomarkers crossing their respective cutoff values. The significance level was set at a *p*-value of less than 0.05.

## 3. Results

The study retrospectively analyzed the background characteristics of children hospitalized with and without pneumonia following SARS-CoV-2 infection. Age and vitamin D levels were compared between the two groups, with children having pneumonia tending to be older (mean ± SD: 6.67 ± 5.01 years) than those without pneumonia (mean ± SD: 5.04 ± 3.95 years), although this difference was not statistically significant (*p* = 0.121). The distribution of children across age categories also did not significantly differ between the groups (*p* = 0.372), with a higher percentage of older children (13–18 years) in the pneumonia group (19.4%) compared to those without pneumonia (7.7%). Similarly, no significant differences were observed in the body mass index (BMI) (*p* = 0.742) and vitamin D levels (*p* = 0.110) between the groups.

Significantly higher Pediatric Pneumonia Ultrasound (PedPne US) scores were observed in children with pneumonia both at initial assessment and after 7 days of hospitalization. Initially, the mean PedPne US score was substantially higher in the pneumonia group (3.89 ± 1.08) compared to the no pneumonia group (1.93 ± 0.27), with this difference reaching statistical significance (*p* < 0.001). Similarly, after 7 days, children with pneumonia showed a significant increase in the mean PedPne US score (0.99 ± 0.74) compared to those without pneumonia (0.21 ± 0.43), again demonstrating a significant association (*p* < 0.001), as presented in Table 1.

Significant elevations were observed in CRP levels (44.2 ± 51.4 mg/L vs. 18.1 ± 18.1 mg/L, *p* = 0.006), procalcitonin (2.47 ± 1.51 ng/mL vs. 0.69 ± 0.70 ng/mL, *p* < 0.001), interleukin-6 (IL-6) (6.75 ± 6.59 pg/mL vs. 1.43 ± 1.77 pg/mL, *p* < 0.001), and ferritin (80.85 ± 73.51 ng/mL vs. 51.44 ± 33.54 ng/mL, *p* = 0.020). White blood cell (WBC) count, neutrophil percentage, neutrophil-to-lymphocyte ratio (NLR), and derived NLR (dNLR) also showed significant increases in the pneumonia group. The erythrocyte sedimentation rate (ESR) was notably higher in the pneumonia group (22.4 ± 13.4 mm/h) compared to those without pneumonia (11.1 ± 5.4 mm/h), with a *p*-value less than 0.001.

COVID-19-specific immunoglobulin M (IgM) levels were considerably higher in children with pneumonia (147.6 ± 75.8 IU/mL) compared to those without (10.9 ± 52.1 IU/mL, *p* < 0.001), indicating a more recent infection; the levels of COVID-19-specific immunoglobulin G (IgG) did not differ significantly (*p* = 0.255). Additionally, significant differences were noted in the levels of IgA (*p* = 0.028) and IgE (*p* = 0.002) (Table 2).

CRP showed a significant increase in patients with post-COVID-19 pneumonia (9.57 ± 9.02 mg/L vs. 4.85 ± 2.80 mg/L, *p* = 0.046) as well as interleukin-6 (IL-6) levels that were substantially elevated in the pneumonia group (6.75 ± 6.59 pg/mL vs. 1.43 ± 1.77 pg/mL, *p* < 0.001). The erythrocyte sedimentation rate significantly increased in patients with pneumonia (12.92 ± 6.90 mm/h) compared to those without (8.43 ± 3.41 mm/h), with a *p*-value of less than 0.001.

COVID-19-specific immunoglobulin G and M showed a significant increase (*p* < 0.001 for both). This pattern was echoed in the general IgG levels, which were significantly higher in children with pneumonia (810.9 ± 290.6 mg/dL vs. 760.5 ± 200.3 mg/dL, *p* < 0.001), whereas IgM levels did not differ significantly (*p* < 0.001). Additionally, dNLR—an indicator of systemic inflammation—was higher in those with pneumonia (1.09 ± 2.63 vs. 0.73 ± 1.65, *p* = 0.003). Conversely, there was an increase in ferritin levels (80.85 ± 73.31 ng/mL vs. 51.44 ± 33.54 ng/mL, *p* = 0.002) and a significant change in IgE levels (*p* = 0.029) (Table 3).

The subgroup analysis in Table 4 of children with pneumonia reveals significant differences in certain inflammatory markers across age categories. Notably, while the initial PedPne scores did not show significant differences (*p* = 0.084), the analysis highlighted a substantial variation in the white blood cell (WBC) counts (*p* = 0.002) and ferritin levels (*p* = 0.001), suggesting age-related differences in the immune response to pneumonia. Specifically, WBC counts were highest in the 7–13 year age group. Ferritin also showed significant variation, which may reflect different inflammatory or infection processes in pediatric pneumonia across different age groups.

In the evaluation of best cutoff values for predicting pneumonia development post-SARS-CoV-2 infection, significant findings were observed for several laboratory parameters, which were analyzed for their diagnostic efficacy. The dNLR, IL-6, and PedPne scores demonstrated high diagnostic accuracy. Specifically, dNLR exhibited a cutoff value of 1.88, with a sensitivity of 77.0% and specificity of 85.7%, achieving an AUC of 0.802 with a statistically significant *p*-value of less than 0.001. Similarly, IL-6 was identified as a robust biomarker with a cutoff of 6.1 pg/mL, which achieved a sensitivity of 70.3% and a specificity of 92.9%, with an AUC of 0.869, also showing a *p*-value of less than 0.001.

**Figure 1:**
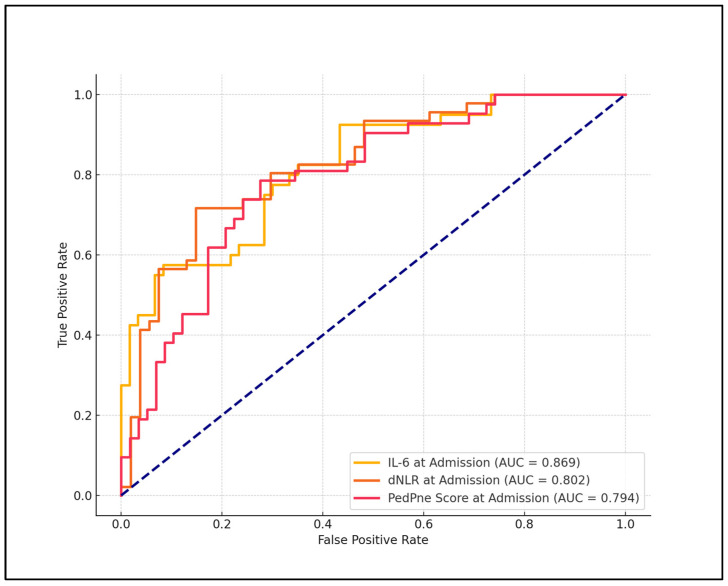
ROC analysis for pneumonia development among children admitted with COVID-19.

Furthermore, the PedPne ultrasound score, which directly assessed pneumonia severity, was set at a cutoff of 3.3, yielding a sensitivity of 75.7% and a specificity of 78.6%, with an AUC of 0.794, indicating high predictive power (*p* < 0.001). In contrast, the neutrophil-to-lymphocyte ratio (NLR) displayed a lower predictive capability with a cutoff of 3.84, sensitivity of 52.7%, specificity of 57.1%, and an AUC of 0.485, coupled with a non-significant *p*-value of 0.327, as presented in Table 5 and Figure 1.

**Figure 2:**
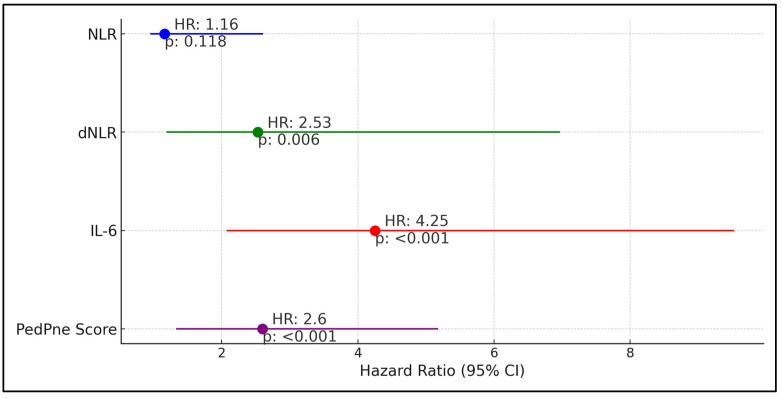
Regression analysis for pneumonia development among children admitted for COVID-19.

dNLR showed a hazard ratio (HR) of 2.53 with a confidence interval (CI) ranging from 1.19 to 6.97 and a *p*-value of 0.006, indicating a more than a twofold increase in the risk of pneumonia when dNLR values were above the cutoff. Similarly, IL-6 emerged as a strong predictor, with a hazard ratio of 4.25 (CI: 2.07–9.53) and a *p*-value of less than 0.001, suggesting a fourfold increase in pneumonia risk. Additionally, the PedPne score, which evaluates the ultrasound findings indicative of pneumonia, had a hazard ratio of 2.60 (CI: 1.33–5.18) with a *p*-value of less than 0.001, reinforcing its utility in predicting pneumonia development. In contrast, the neutrophil-to-lymphocyte ratio (NLR) displayed a hazard ratio of 1.16 (CI: 0.95–2.61) with a *p*-value of 0.118, indicating that while there was an observed increase in risk, it was not statistically significant, as described in Table 6 and Figure 2.

## 4. Discussion

### 4.1. Analysis of Findings

This study delineated the prognostic utility of Pediatric Pneumonia Ultrasound Scores (PedPne) and specific inflammatory biomarkers like dNLR and IL-6 in pediatric patients developing pneumonia post-SARS-CoV-2 infection. The significant correlation found between high PedPne scores and the progression of pneumonia underscores the importance of ultrasound evaluations in clinical settings for early detection and management. Notably, the study’s findings indicate that elevations in dNLR and IL-6 not only signify an active inflammatory response but are also predictive of pneumonia outcomes, aligning with emerging literature that supports their roles as critical markers of systemic inflammation and disease severity in viral infections.

The derivation of optimal cutoff values for these markers provided objective criteria to aid in the clinical decision-making process. For instance, dNLR showed a high specificity and sensitivity (85.7% and 77.0%, respectively), suggesting that it can reliably distinguish between pediatric patients at higher risk for pneumonia from those with less severe manifestations. Similarly, IL-6’s cutoff value at 6.1 pg/mL, demonstrating an AUC of 0.869, highlighted its strong predictive power, consistent with its recognized role in orchestrating prolonged inflammatory responses in viral pneumonias.

Regression analysis further substantiated the predictive strength of these biomarkers, with IL-6 showing substantial hazard ratios, indicating a significant increase in the likelihood of pneumonia development as these marker levels rise. PedPne score correlated with inflammation, and insights into the inflammatory dynamics allow for a more nuanced understanding and anticipation of disease progression, potentially guiding targeted therapeutic interventions and monitoring strategies.

In the development and application of a lung ultrasound score, Ciuca et al. [8] first introduced this novel diagnostic tool, finding a very strong positive correlation between the PedPne score and inflammatory markers such as C-reactive protein and erythrocyte sedimentation rate among 64 patients diagnosed from 217 that were screened. A median lung ultrasound score of 8.02 aligned closely with radiographic findings, confirming its reliability. Subsequently, in 2022, the same group published an extensive exhaustive score, named the PedPne score, and expanded the utility across a larger cohort of 321 screened patients with 81 confirmed cases of pneumonia, demonstrating its consistent application over the disease course with strong correlations noted between the US score and CRP, procalcitonin, and leukocyte counts across multiple evaluations [20]. These findings underscore the clinical relevance of the PedPne score as a robust, non-invasive measure for both the diagnosis and ongoing monitoring of pediatric pneumonia.

In a randomized clinical trial by Guitart et al. [20], the integration of a lung ultrasound and procalcitonin markedly improved the management of pediatric bacterial pneumonia by guiding antibiotic use and reducing unnecessary radiation exposure, demonstrating that when LUS and PCT values were below a set threshold, antibiotic use could be safely reduced, evidenced by a 77% decrease in chest X-rays and no significant cost increase. This method contrasts with findings from Bazdyrev et al. [21], who explored the long-term management of post-COVID-19 organizing pneumonia (PCOP), noting that up to 25% of patients required extended treatment with corticosteroids, fraught with potential relapses and severe side effects. The juxtaposition of these studies highlights the evolving understanding and management strategies in pediatric pneumonia—from acute care optimizations to addressing chronic post-viral effects—underscoring the critical need for precise diagnostic and therapeutic approaches in diverse clinical scenarios.

Indolfi et al. [22] and Ciuca et al. [23] provided significant insights into the utility of lung ultrasound in pediatric respiratory conditions post-COVID-19 and pneumonia, respectively. Indolfi et al. focused on the long-term respiratory complications associated with COVID-19 in children, finding that 27.0% of the 104 children evaluated showed positive LUS findings, indicating persistent pulmonary abnormalities. This study, which noted a significant association of a higher body mass index with worse COVID-19 symptoms and LUS scores (*p* < 0.05), highlights the potential of LUS in monitoring long COVID effects in pediatric patients. On the other hand, Ciuca et al. demonstrated the effectiveness of LUS over chest X-ray in diagnosing consolidated pneumonia in children, with LUS showing superior sensitivity. They reported an almost perfect agreement between LUS and CXR with a Cohen’s kappa coefficient of K = 0.89 ± 0.04 SD, *p* = 0.000. Both studies underline LUS as a valuable diagnostic tool, with Indolfi et al. emphasizing its role in long-term management of post-COVID-19 complications and Ciuca et al. confirming its higher diagnostic accuracy for acute pneumonia, suggesting a shift towards more widespread use of LUS in pediatric respiratory assessments.

The findings of this study introduce novel insights into the clinical utility of combining PedPne with inflammatory biomarkers such as dNLR and IL-6 for diagnosing and managing pneumonia in pediatric patients post-SARS-CoV-2 infection. This approach offers a robust predictive framework, enhancing early diagnostic capabilities and potentially guiding tailored therapeutic strategies. The demonstrated sensitivity and specificity of these biomarkers, especially in a pediatric population, signify a significant advancement in our ability to not only detect but also prognosticate the course of pneumonia following COVID-19, providing a valuable tool in the clinical arsenal against the variable presentations of the SARS-CoV-2 infection in children.

In understanding the linkage between PedPne scores and inflammatory biomarkers with pneumonia progression, it becomes evident that these metrics can provide critical insights into disease severity and trajectory. Elevated WBC counts and ferritin levels, as noted in specific age groups, could signify more intense inflammatory responses, which correlate with increased disease severity. Clinically, incorporating these biomarkers into routine diagnostic protocols could enhance the early detection and stratification of pneumonia severity, guiding more tailored treatment approaches. For instance, higher PedPne scores alongside elevated ferritin might prompt earlier initiation of anti-inflammatory treatments. By integrating such biomarkers systematically, clinicians can better predict patient outcomes, optimize resource allocation, and potentially reduce hospital stay durations through more precise management strategies.

### 4.2. Study Limitations and Future Perspectives

The sample size, though sufficient to achieve statistical significance, was relatively small, which might limit the generalizability of the findings. The observational nature of the study also restricts the ability to infer causality between the biomarkers and pneumonia development. Furthermore, the study relied on data from a single pediatric center, which might introduce center-specific biases in patient management and diagnostic criteria that could affect the study’s outcomes. The study’s limitations include potential biases from sample size and demographic homogeneity, which may impact the generalizability of the findings. Future research should focus on including a broader range of biomarkers and expanding to multi-center studies to enhance the robustness and applicability of the results.

## 5. Conclusions

In conclusion, this study validated the significant predictive value of PedPne scores, dNLR, and IL-6 in identifying and managing pediatric pneumonia post-COVID-19, with these tools demonstrating high specificity and sensitivity in clinical assessments. The integration of these biomarkers into routine clinical protocols could potentially enhance the diagnostic accuracy and therapeutic management of pediatric pneumonia, leading to improved patient outcomes. Future studies with larger, multi-center cohorts are recommended to further substantiate these findings and refine the predictive models for broader clinical application.

## Figures and Tables

**Table 1:** Background characteristics of children with and without pneumonia during COVID-19 hospitalization.

Variables	No Pneumonia (*n* = 28)	Pneumonia (*n* = 38)	*p*-Value
Age, years (mean ± SD)	5.04 ± 3.95	6.67 ± 5.01	0.121
Age category			0.372
1–6 years	15 (53.8%)	17 (47.2%)	
7–12 years	11 (38.5%)	12 (33.3%)	
13–18 years	2 (7.7%)	7 (19.4%)	
BMI (mean ± SD)	17.01 ± 1.94	16.63 ± 3.05	0.742
Vitamin D (mean ± SD)	19.65 ± 6.11	20.52 ± 6.64	0.110
PedPne US initial score (mean ± SD)	1.93 ± 0.27	3.89 ± 1.08	<0.001
PedPne US 7-day score (mean ± SD)	0.21 ± 0.43	0.99 ± 0.74	<0.001

SD—standard deviation; BMI—body mass index; PedPne—pediatric pneumonia; US—ultrasound.

**Table 2:** Comparison of laboratory findings of children with and without pneumonia during COVID-19 hospitalization (initial measurements).

Variables (Mean ± SD)	Normal Range	No Pneumonia (*n* = 28)	Pneumonia (*n* = 38)	*p*-Value
Inflammatory markers				
ESR	0–20 mm/h	11.1 ± 5.4	22.4 ± 13.4	<0.001
C-reactive protein	<10 mg/L	18.1 ± 18.1	44.2 ± 51.4	0.006
WBC	4.0–10.0 × 10^9^/L	12.5 ± 5.0	14.8 ± 5.9	0.004
Neutrophil %	40–60%	51.0 ± 24.4	62.0 ± 22.3	0.048
Lymphocyte %	20–40%	17.6 ± 17.0	23.0 ± 20.2	0.363
Procalcitonin	<0.5 ng/mL	0.69 ± 0.70	2.47 ± 1.51	<0.001
IL-6	<7 pg/mL	1.43 ± 1.77	6.75 ± 6.59	<0.001
Ferritin	30–400 ng/mL	51.44 ± 33.54	80.85 ± 73.51	0.020
NLR	-	5.36 ± 9.33	8.80 ± 11.17	0.008
dNLR	-	0.73 ± 1.65	1.49 ± 2.63	0.006
Antibody count				
COVID-19-specific Immunoglobulin G	-	115.1 ± 81.7	154.4 ± 127.0	0.255
COVID-19-specific Immunoglobulin M	-	10.9 ± 52.1	147.6 ± 75.8	<0.001
IgG	-	551.7 ± 199.1	795.6 ± 297.7	0.238
IgM	-	123.2 ± 64.3	207.9 ± 53.1	<0.001
IgA	-	86.6 ± 29.0	100.3 ± 96.4	0.028
IgE	-	169.3 ± 284.0	82.2 ± 161.0	0.002

SD—standard deviation; WBC—white blood cell; dNLR—derived neutrophil-to-lymphocyte ratio; IL-6—interleukin 6; ESR—erythrocyte sedimentation rate.

**Table 3:** Comparison of laboratory findings of children with and without pneumonia during COVID-19 hospitalization (7-day measurements).

Variables (Mean ± SD)	Normal Range	No Pneumonia (*n* = 28)	Pneumonia (*n* = 38)	*p*-Value
Inflammatory markers				
ESR	0–20 mm/h	8.43 ± 3.41	12.92 ± 6.90	<0.001
C-reactive protein	<10 mg/L	4.85 ± 2.80	9.57 ± 9.02	0.046
WBC	4.0–10.0 × 10^9^/L	12.51 ± 5.02	14.77 ± 5.89	0.058
Neutrophil %	40–60%	56.01 ± 24.44	61.97 ± 22.27	0.027
Lymphocyte %	20–40%	17.61 ± 17.08	23.04 ± 20.21	0.530
Procalcitonin	<0.5 ng/mL	0.69 ± 0.70	2.47 ± 11.51	0.408
IL-6	<7 pg/mL	1.43 ± 1.77	6.75 ± 6.59	<0.001
Ferritin	30–400 ng/mL	51.44 ± 33.54	80.85 ± 73.31	0.002
NLR	-	8.36 ± 9.33	8.80 ± 11.17	0.628
dNLR	-	0.73 ± 1.65	1.09 ± 2.63	0.003
Antibody count				
COVID-19-specific Immunoglobulin G	-	130.1 ± 80.5	266.3 ± 120.7	<0.001
COVID-19-specific Immunoglobulin M	-	45.2 ± 30.1	150.7 ± 75.4	<0.001
IgG	-	760.5 ± 200.3	810.9 ± 290.6	<0.001
IgM	-	120.4 ± 65.1	115.3 ± 55.2	<0.001
IgA	-	88.2 ± 30.5	102.7 ± 98.2	0.564
IgE	-	170.2 ± 285.0	85.3 ± 162.5	0.029

SD—standard deviation; WBC—white blood cell; dNLR—derived neutrophil-to-lymphocyte ratio; IL-6—interleukin 6; ESR—erythrocyte sedimentation rate.

**Table 4:** Subgroup analysis of PedPne scores and inflammatory markers by age categories of children with pneumonia.

Variables (Mean ± SD)	1–6 Years (*n* = 32)	7–13 Years (*n* = 23)	13–18 Years (*n* = 9)	*p*-Value
Initial PedPne score	3.58 ± 0.32	3.46 ± 0.16	3.72 ± 0.37	0.084
7-day PedPne score	0.94 ± 0.36	0.87 ± 0.38	0.74 ± 0.32	0.293
ESR	11.14 ± 6.19	10.98 ± 6.47	11.57 ± 5.40	0.365
C-reactive protein	9.87 ± 9.43	9.16 ± 7.37	8.74 ± 8.17	0.093
WBC	14.28 ± 6.39	15.52 ± 5.18	13.67 ± 5.35	0.002
Neutrophil %	68.21 ± 22.26	62.67 ± 20.18	67.89 ± 21.28	0.297
Lymphocyte %	21.74 ± 21.37	23.39 ± 20.21	22.26 ± 20.39	0.831
Procalcitonin	3.23 ± 6.17	2.12 ± 10.46	2.07 ± 8.30	0.007
IL-6	1.52 ± 5.31	1.68 ± 7.44	1.83 ± 6.22	0.067
Ferritin	80.47 ± 70.19	73.89 ± 73.23	77.32 ± 71.21	0.001
NLR	8.21 ± 9.31	7.89 ± 8.43	8.53 ± 10.37	0.144
dNLR	1.92 ± 2.16	1.63 ± 1.44	1.29 ± 2.50	0.758

SD—standard deviation; WBC—white blood cell; dNLR—derived neutrophil-to-lymphocyte ratio; IL-6—interleukin 6; ESR—erythrocyte sedimentation rate.

**Table 5:** Best cutoff values for pneumonia development.

Laboratory Parameter	Best Cutoff Value	Sensitivity	Specificity	AUC	*p*-Value
NLR	3.84	52.7%	57.1%	0.485	0.327
dNLR	1.88	77.0%	85.7%	0.802	<0.001
IL-6	6.1 pg/mL	70.3%	92.9%	0.869	<0.001
PedPne Score	3.3	75.7%	78.6%	0.794	<0.001

dNLR—derived neutrophil-to-lymphocyte ratio; AUC—Area Under Curve.

**Table 6:** Regression analysis for pneumonia development among children admitted for COVID-19.

Factors above the Best Cutoff	Hazard Ratio	95% CI	*p*-Value
NLR	1.16	0.95–2.61	0.118
dNLR	2.53	1.19–6.97	0.006
IL-6	4.25	2.07–9.53	<0.001
PedPne Score	2.60	1.33–5.18	<0.001

dNLR—derived neutrophil-to-lymphocyte ratio; CI—confidence interval.

## Data Availability

The data presented in this study are available on request from the corresponding author.
